# Microbial taxonomical composition in spruce phyllosphere, but not community functional structure, varies by geographical location

**DOI:** 10.7717/peerj.7376

**Published:** 2019-07-19

**Authors:** Yunshi Li, Xiukun Wu, Wanfu Wang, Minghao Wang, Changming Zhao, Tuo Chen, Guangxiu Liu, Wei Zhang, Shiweng Li, Huaizhe Zhou, Minghui Wu, Ruiqi Yang, Gaosen Zhang

**Affiliations:** 1 Key Laboratory of Desert and Desertification, Northwest Institute of Eco-Environment and Resources, Chinese Academy of Sciences, Lanzhou, China; 2 University of Chinese Academy of Sciences, Beijing, China; 3 Key Laboratory of Extreme Environmental Microbial Resources and Engineering, Gansu Province, Lanzhou, China; 4 Conservation Institute, Dunhuang Academy, Dunhuang, China; 5 State Key Laboratory of Grassland Agro-Ecosystems, School of Life Sciences, Lanzhou University, Lanzhou, China; 6 State Key Laboratory of Cryospheric Sciences, Northwest Institute of Eco-Environment and Resources, Chinese Academy of Sciences, Lanzhou, China; 7 Lanzhou Jiaotong University, School of Environmental and Municipal Engineering, Lanzhou, China; 8 National University of Defense Technology, College of Computer, Changsha, China

**Keywords:** Microbial taxonomical composition, Community functional structure, Geographical location, Phyllosphere, Functional redundancy, *Picea* spp

## Abstract

Previous studies indicate that the plant phenotypic traits eventually shape its microbiota due to the community assembly based on the functional types. If so, the distance-related variations of microbial communities are mostly only in taxonomical composition due to the different seeds pool, and there is no difference in microbial community functional structure if the location associated factors would not cause phenotypical variations in plants. We test this hypothesis by investigating the phyllospheric microbial community from five species of spruce (*Picea* spp.) trees that planted similarly but at three different locations. Results indicated that the geographical location affected microbial taxonomical compositions and had no effect on the community functional structure. In fact, this actually leads to a spurious difference in the microbial community. Our findings suggest that, within similar host plants, the phyllosphere microbial communities with differing taxonomical compositions might be functionally similar.

## Introduction

The phyllosphere provides an ecological niche for microorganisms inhabiting it (Delmotte et al., 2009; Rico et al., 2014; Leveau, 2015). These taxonomically structured microbial communities are functionally diverse and play an important role for the plant ecological strategies aboveground (Friesen et al., 2011; Berlec, 2012; Kembel et al., 2014; Rosado et al., 2018). For instance, the microbial members are involved in disease resistance and/or pathogenesis of plant, promoting plant growth and development, changing plant foliar activity, and fixing nitrogen (Innerebner, Knief & Vorholt, 2011; Reed, Cleveland & Townsend, 2011). To this date, numerous investigations have shed light on the phylogenetic compositions of microbial life within plant phyllosphere. These studies have revealed that compositional changes within microbial functional groups are correlated to changes in phyllosphere ecosystem processes (Cordier et al., 2012; Remus-Emsermann et al., 2012; Rastogi, Coaker & Leveau, 2013). However, they did not demonstrate a relationship between microbial taxonomical compositions and community functional structures across geographical locations.

Several studies have compared taxonomic structure of phyllosphere microbial communities from the same tree species across a range of geographic locations. Therefore, we can infer that geographic locations may or may not influence the structure of these taxonomic microbial communities. For example, microbial phyllosphere communities on different *Tamarix* species are highly similar in the same locale, whereas trees of the same species that grow in different regions possess distinct microbial communities (Finkel et al., 2011). However, there was a remarkably little influence of geographic location on phyllosphere community composition of *Pinus ponderosa* leaves, even over thousands of kilometers (Redford et al., 2010). Studies further speculated that location-dependent differences observed in the leaf microbiome were between samples taken from distant locations with very different climates and soil properties (Badri et al., 2013). The taxonomical microbial community shifts among various locations could be due to location-specific properties but may also reflect host plant species relatedness. In general, the phyllosphere microbiota is a multilayered structure, composed of both a flexible pool of microbes modulated by the environment and a microbiota under the host genotypic and phenotypic control.

Host species are known to influence the taxonomical composition of phyllosphere microbial communities. Current knowledge indicates two basic mechanisms that possibly explain the host species effect. First, plant genotype may vary the phyllosphere microbial community (Whipps et al., 2008; Bálint et al., 2013; Bodenhausen et al., 2014). It has now been demonstrated that within plant species, contrasting genotypes can support different microbial communities. Second, plant phenotypic differentiation has also been shown to contribute to the variation of phyllosphere microbial communities. Plasticity basis of variations in the phenotype of individual plants caused basic differences in phyllosphere habitat, in which epiphytic microbes will choose favorable sites for survival and growth (Hunter et al., 2010; Laforest-Lapointe, Messier & Kembel, 2016). Further, microbial specialization to substrates probably underlies a fraction of differences in the phyllosphere microbiota, as bacterial species partition the niche space according to their substrate preferences and use (Bulgarelli et al., 2013). As a result, leaf exudates and the response of microorganisms to those as well as to leaf morphology were shown to alter the relative abundances of the taxa that are present (Reisberg et al., 2013; Bodenhausen et al., 2014).

In a recent report, we showed that the plant phenotypic traits eventually shape its microbiota due to the community assembly based on the functional types (Li et al., 2018). Plant trait differences across geographical locations had both genetic and plasticity bases, with a stronger contribution of the latter (Schlichting, 1986; Al Hayek et al., 2014). In different environments, a given genotype expresses different phenotypes, a phenomenon known as phenotypic plasticity (Sultan, 2000; Nicotra et al., 2010). For example, we can always observe the variations of tree morphology, physiology, and life history under different conditions. Phenotypic variations within a plant species can affect microbial communities due to their varying traits. Most changes in microbial communities occurred as results of differing phenotypes under similar environmental conditions. After excluding plant effects, the dissimilarity in the microbial taxonomical composition come from the different regional species pool (Kinkel, 1997; Müller et al., 2016). Callens et al. (2018) confirmed that the differences in the environmental pool of colonizers can influence microbiota community assembly on the host. Previous findings indicated that geographic locations will lead to an indirect effect on the microbial community functional structure. Therefore, if the location associated factors do not cause phenotypical variations in plants, the plant-associated microbial communities will only differ in taxonomical compositions, rather than functional structure variations. This is also justified based upon the high species diversity in microbial communities and the ability of microbes to adapt rapidly to new conditions. Dissimilarities in the community functional structures only result from the plant per se when under similar environmental conditions. To test this assumption, we compared the microbial community dissimilarities of the same tree species sampled from different geographical locations, to those of different tree species from the same site. We have done this by sampling trees in different areas having relatively uniform climatic conditions. This paper tries to find a reasonable answer to the following questions: how do the microbial communities reflect geographical location variations? Do such alterations have consequences on microbial taxonomical compositions or community functional structures? If geographic location-governed factors do not cause phenotypic variations in plants, is there a variation in the community functional structure that associates with different geographical locations?

## Materials and Methods

### Sample sites and sampling

**Figure 1:**
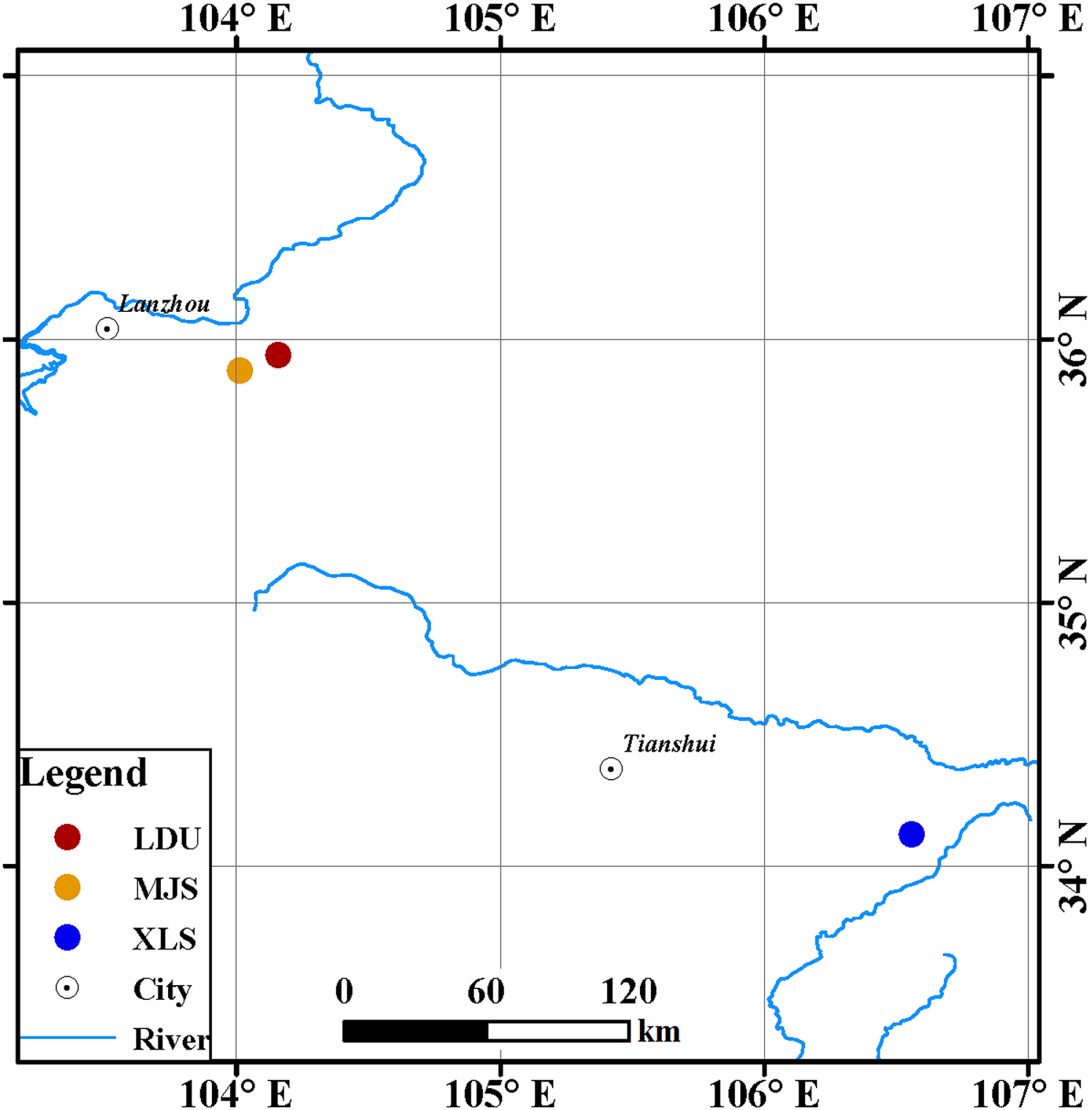
Geographic locations of spruce trees sampled. The abbreviations of study sites are as follows: LDU, Yuzhong campus of Lanzhou University in Lanzhou city; MJS, a site in the Xinglongshan forest in Majiasi; XLS, Xiaolongshan forest farm in Tianshui City.

Leaves of spruce (*Picea* spp.) tree species were collected from three sites in Gansu Province, China (Fig. 1): in the Yuzhong campus of Lanzhou University in Lanzhou city (LDU, 104.16°E, 35.94°N), in Xiaolongshan forest farm in Tianshui City (XLS, 106.56°E, 34.12°N), and a site in the Xinglongshan forest in Majiasi (MJS, 104.01°E, 35.88°N). At each location, five spruce (*Picea* spp.) tree species of *Picea abies*, *Picea crassifolia*, *Picea koraiensis*, *Picea likiangensis var. rubescens*, and *Picea wilsonii* were selected. In total, 15 leaf samples were obtained at each location (three replicates per tree).

In the year 2006, spruce seedlings were bred in Xiaolongshan forest farm. The spruce species germinated from seeds, which were surface sterilized with a 0.50% potassium permanganate (KMnO_4_) solution. Then the spruce seedlings were transplanted in the Yuzhong campus of Lanzhou University and the Xinglongshan forest in Majiasi in the year 2009 and 2014, respectively. All samples were collected in early October 2017 and all the trees were 11 years old. Weather conditions were warm and moist during the sampling.

The Yuzhong campus of Lanzhou University is in the northwest of the Loess Plateau, the middle part of Gansu Province, which is 46 km from Lanzhou city. The climate of this location is semiarid, and the mean annual precipitation is low (381.80 mm), nearly 78% of rainfall events are below 10 mm and 36% of precipitations are below five mm, and the mean annual temperature is 6.57 °C and droughts commonly occur. The soil in the region, which is two m deep, is the clay loam of Loess origin with a bulk density ranging from 1.38 to 1.45 g cm^−3^, and the water holding capacity of the soil is 21.18%. The results are supported by Wang et al. (2008).

The Xinglongshan forest in Majiasi is a unique island-like area where primitive forest is well preserved at the western edge of the Chinese Loess Plateau. The mean annual temperature is 4.10 °C, with a mean annual precipitation of 625 mm (Lei et al., 2010). Leached brown soil covers the area and was developed under semiarid climate under forest cover (Liu et al., 1990).

Xiaolongshan forest farm is located in the southeast of Gansu Province. The climate of this location is warm with humid and semi-humid continental monsoon. It is characterized by a long-term average annual temperature of 7–12 °C and an average annual precipitation of 600–900 mm. The soil in the area is dominated by mountain cinnamon soils and brown forest soils listed in the Chinese Soil Taxonomy. The information was obtained from the Xiaolongshan Forestry Bureau of Gansu Province.

Only “healthy” leaves that with no signs of disease or decay (such as browning or spotting) were selected assessed with the naked eye. Ten grams of fresh needles and shoots were collected from multiple canopy positions and mixed into a 100 ml plastic centrifuge tube containing 50 ml sterile phosphate buffer. The tube was agitated on vortex for 10 s and repeated for six times, and then the buffer was filtered through an EMD Millipore Sterivex-GV Polyvinylidene Fluoride 0.22 µm filter (Millipore, Billerica, MA, USA). Then, the filter was placed in a sterile screw-cap tube and frozen at −80 °C before processing.

### DNA extraction, PCR amplification, and sequencing processing

The phylloshperic microbial DNA was extracted using the E.Z.N.A.^®^ Soil/Stool DNA Kit (Omega Bio-tek, Norcross, GA, USA) according to manufacturer’s protocols. Quantity and quality of the genomic DNA were checked on 0.80% (w/v) agarose gels. The V4 hypervariable region of the bacterial 16S rRNA gene was amplified with TransGen AP221-02 (TransStart FastPfu DNA Polymerase) using primers 515F-806R (Caporaso et al., 2011); PCRs contained 4 µl 5 × FastPfu buffer, two µl 2.50 mM deoxyribonucleoside triphosphates mix, 0.8 µl forward primer (five μM), 0.8 µl reverse primer (five μM), 0.40 µl FastPfu Polymerase, 0.20 µl 20 mg/mL bovine serum albumin solution and 10 ng Template DNA in a volume of 20 µl. The ITS1-ITS2 (White et al., 1990) was used to target partial fragments of the fungal ITS gene with TAKARA rTaq DNA polymerase (Takara Shuzo, Kyoto, Japan); PCRs contained 2 µl 10 × PCR buffer, 2 µl 2.50 mM deoxyribonucleoside triphosphates mix, 0.8 µl forward primer (five μM), 0.80 µl reverse primer (five μM), 0.2 µl rTaq Polymerase, 0.20 µl 20 mg/ml bovine serum albumin solution and 10 ng Template DNA in a volume of 20 µl. All samples were heated at 94 °C for 3 min in the first round of denaturation and were then subjected to 32 cycles of PCR consisting of 30 s at 95 °C, 30 s at 62 °C, and 45 s at 72 °C. Cycling was performed in an automated DNA thermal cycler (ABI GeneAmp^®^ PCR System 9700). After the last cycle, the samples were incubated for an additional 10 min at 72 °C. Amplicons were extracted from 2% (w/v) agarose gels and purified using the AxyPrep DNA Gel Extraction Kit (Axygen Biosciences, Union City, CA, USA) according to the manufacturer’s instructions and quantified using QuantiFluor™ -ST (Promega, Madison, WI, USA). Sample libraries were pooled in equimolar and paired-end sequenced (2 × 300 bp) on an Illumina MiSeq (Illumina, San Diego, CA, USA) platform. Raw FASTQ files were quality-filtered by Trimmomatic and merged by FLASH with the following criteria: (i) The reads were truncated at any site receiving an average quality score <20 over a 50 bp sliding window. (ii) Sequences whose overlap being longer than 10 bp were merged according to their overlap with mismatch no more than two bp. (iii) Sequences of each sample were separated according to barcodes (exactly matching) and Primers (allowing for two nucleotides mismatching) and reads containing ambiguous bases were removed. Then the dataset was analyzed using QIIME (version 1.9.1, http://www.qiime.org). Operational taxonomic units (OTUs) with 97% similarity cutoff were clustered using UPARSE (version 7.1, http://drive5.com/uparse/) (Edgar, 2013). Chimeric sequences were identified and removed using UCHIME (Edgar et al., 2011). The taxonomy of each bacterial 16S rRNA gene sequence and fungal ITS sequence was analyzed by the RDP Classifier algorithm (http://rdp.cme.msu.edu/) against the Silva (Release119, http://www.arb-silva.de) and UNITE (Release 7.0, http://unite.ut.ee/index.php) databases at a confidence threshold of 70%, respectively. Sequences shorter or longer than the expected amplicons size and chimeras were removed. Cyanobacteria, mitochondria, archaeal and non-fungal reads were also filtered. After quality control, 1,921,840 and 2,915,075 valid sequences were clustered into 3,588 bacterial and 3,694 fungal OTUs at 97% sequence identity level, respectively (Table S1). There are 40,654 and 120 bacterial and fungal sequences could not be classified to any known bacterial and fungal phyla, respectively. Here, we defined OTUs with abundance higher than 5% in at least one of the replicates as abundant OTUs. The rarefaction analysis based on Mothur v.1.21.1 software was conducted to reveal the diversity indices, including the Chao1, Shannon, Simpson, and coverage indices. All sequences can be downloaded from the NCBI Sequence Read Archive database under the accession BioProject number PRJNA506625.

### Tax4Fun and FUNGuild analyses

Tax4Fun is an inexpensive method to estimate the functional capacity of a microbial community (Aßhauer et al., 2015). This tool uses 16S rRNA gene profiles and then indirectly infers the abundance of functional genes. The results are KEGG orthologs (KOs) which wholly depend on the Silva ontology. Formatted sequences were clustered at 0.97 sequence similarity described above. Then, taxonomic information was assigned using Silva 123 downloaded from the Tax4Fun website (http://tax4fun.gobics.de/). An OTU table was created and fed to Tax4Fun R package. The Tax4Fun function was run with all default settings as described on the Tax4Fun website.

The fungal functional guild of the OTUs was inferred using FUNGuild v1.0 (http://funguild.org). This tool will infer organism trophic mode (Nguyen et al., 2016).

### Data analysis

Alpha-diversity parameters were estimated (for a complete list of the calculated indexes see Table S2 in Supporting Information), and distance matrices (Bray–Curtis) of the samples were computed. A two-way ANOVA on the calculated alpha-diversity indices were performed in order to test differences in microbiome diversity between the different samples. Variations between different groups in Principal Coordinate Analysis (PCoA) were calculated by permutational multivariate analysis of variance (PERMANOVA) in R package “vegan,” using the function “adonis,” permutations = 999 (R Core Team, 2008). Bray–Curtis distance matrices used in the PCoA analysis were calculated by “dist.shared” command to test differences in the microbiome composition between different samples. A permutational multivariate analysis of dispersion (PERMDISP) for vegan’s betadisper was then run on this Bray–Curtis dissimilarity matrix to test the homogeneity of variance in community data (Anderson & Walsh, 2013). The statistical significance of the relationship between species dissimilarities and geographic locations was tested by ANCOVA analysis using SPSS. Analyses for the Venn diagram generation in microbial communities were performed using the Mothur v.1.21.1 suite of programs. The species typical of the different locations were determined using indicator species analysis (ISA). ISA was performed using the *multipatt* function implemented in the “indicspecies” package in R with 99,999 permutations and allowing combinations between habitats (De Cáceres, Legendre & Moretti, 2010). In this study, ISA were conducted with taxa grouped at the class and order level of taxonomic resolution, respectively. The p.adjust command was used to define a corrected critical *p*-value corresponding to a “false discovery rate” (FDR) of 0.05 (Benjamini & Hochberg, 1995). An FDR of 5% means that among all features called significant, 5% of these are truly null on average. Map created in ArcMap 9.3.

## Results

### Phyllosphere microbial communities associated with spruce trees

To identify the phyllosphere microbiota associated with spruce trees, 16S rRNA and ITS sequences of 45 replicates across five species and three locations were obtained.

A heatmap representation of the abundant OTUs abundances is presented in Fig. S1. A total of 19 abundant OTUs were identified in 16S datasets. A total of 18 identified abundant OTUs were ascribed to four bacterial phyla (Proteobacteria, Actinobacteria, Bacteroidetes, and Firmicutes), while one unclassified at phylum level. A total of 24 abundant OTUs were identified in ITS datasets. At a finer taxonomic level, 20 identified abundant OTUs were ascribed to five fungal orders (Pleosporales, Chaetothyriales, Taphrinales, Capnodiales, and Dothideales), while four were unclassified at the order level. Some OTUs were shared between different species from geographically distant samples, suggesting the associated microbial taxa may be widespread.

In the bacterial communities, the most abundant bacterial phylum was the Proteobacteria, with an average abundance of 54.15% of the reads, followed by Bacteroidetes (20.32%), and Actinobacteria (10.92%). The remaining 14.62% of reads belonged to other bacterial phyla or were unclassified (unable to be taxonomically assigned, given the training set). In the fungal communities, the most abundant fungal order was the Pleosporales, with an average abundance of 33.2% of the reads, followed by Capnodiales (17.1%), Chaetothyriales (8.5%), and Taphrinales (7.8%). The four orders belonged to Ascomycota, which accounted for 66.6% of the reads.

### Community dissimilarity estimation by diversity indices

**Figure 2:**
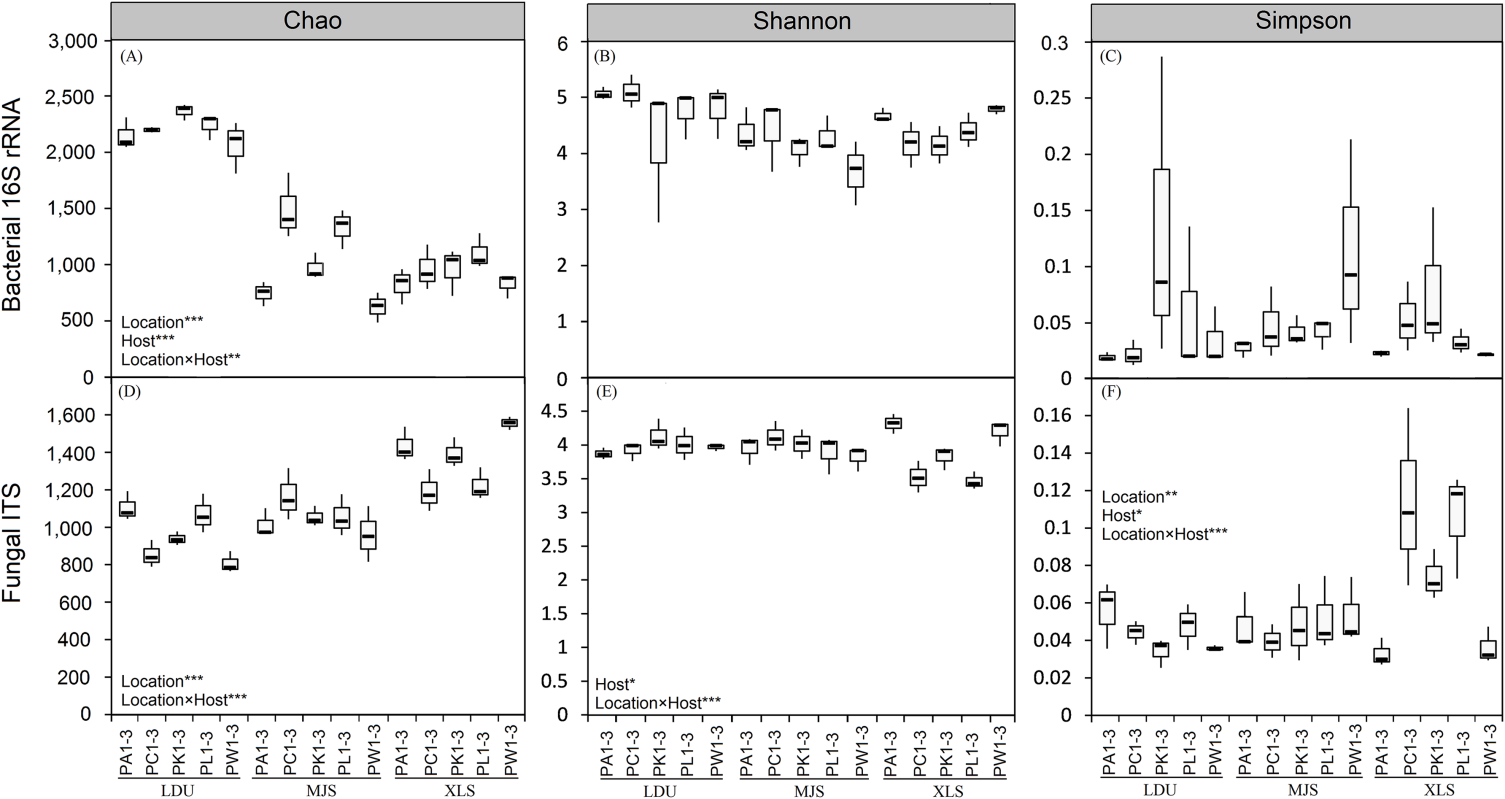
Boxplot of alpha-diversity indecies of bacterial (A, B and C) and fungal (D, E and F) communities among different replicates of phyllosphere samples. Two-way ANOVA on the calculated alpha-diversity indices were performed: *p < 0.05; **p < 0.01; ***p < 0.001. The abbreviations of study sites and plant species are as follows: LDU, Yuzhong campus of Lanzhou University in Lanzhou city; MJS, a site in the Xinglongshan forest in Majiasi; XLS, Xiaolongshan forest farm in Tianshui City. PA, *Picea abies*; PC, *Picea crassifolia*; PK, *Picea koraiensis*; PL, *Picea likiangensis var. rubescens*; PW, *Picea wilsonii*. Number of sample replicates are 3, which are coded as 1–3.

The two-way ANOVA on the alpha-diversity measures (Chao 1 index (*C*), Shannon diversity index (*H*′), and Simpson diversity index (λ); Fig. 2; Table S3) of both bacterial and fungal communities evaluating all the factors considered in the experimental design and their interactions. The results revealed that the geographical location, the host plant species and the interaction of these two factors had the most influence on microbial alpha diversities. Comparing different samples (LDU, MJS, and XLS) in the bacterial communities, only the richness was highly significant with the location effect (*F* = 283.34, *p* < 0.001; Table S3), the host tree effect (*F* = 10.79, *p* < 0.001; Table S3), and the interaction effect of the location and host tree (*F* = 3.97, *p* = 0.003; Table S3), respectively, whereas for the fungal communities, the richness, the evenness, and the diversity were all significant with these factors at different degrees (Table S3). Host tree species had significant, albeit small, influences on the fungal community diversity (*F* = 2.89, *p* < 0.05; Table S3) and evenness (*F* = 3.50, *p* < 0.05; Table S3). The interaction between the sample location and the host tree species showed a highly significant effect of the fungal community for all the analyzed indexes (*C*, *F* = 7.33, *p* < 0.001; *H*′, *F* = 5.57, *p* < 0.001; λ, *F* = 5.04, *p* = 0.001; Table S3), while only the OTUs index was significant in the bacterial community (*D*, *p* < 0.001; *H*′, *p* = 0.78; λ, *p* = 0.15; Table S3).

### Similarity in community composition depends upon taxonomical OTUs and functional groups

**Figure 3:**
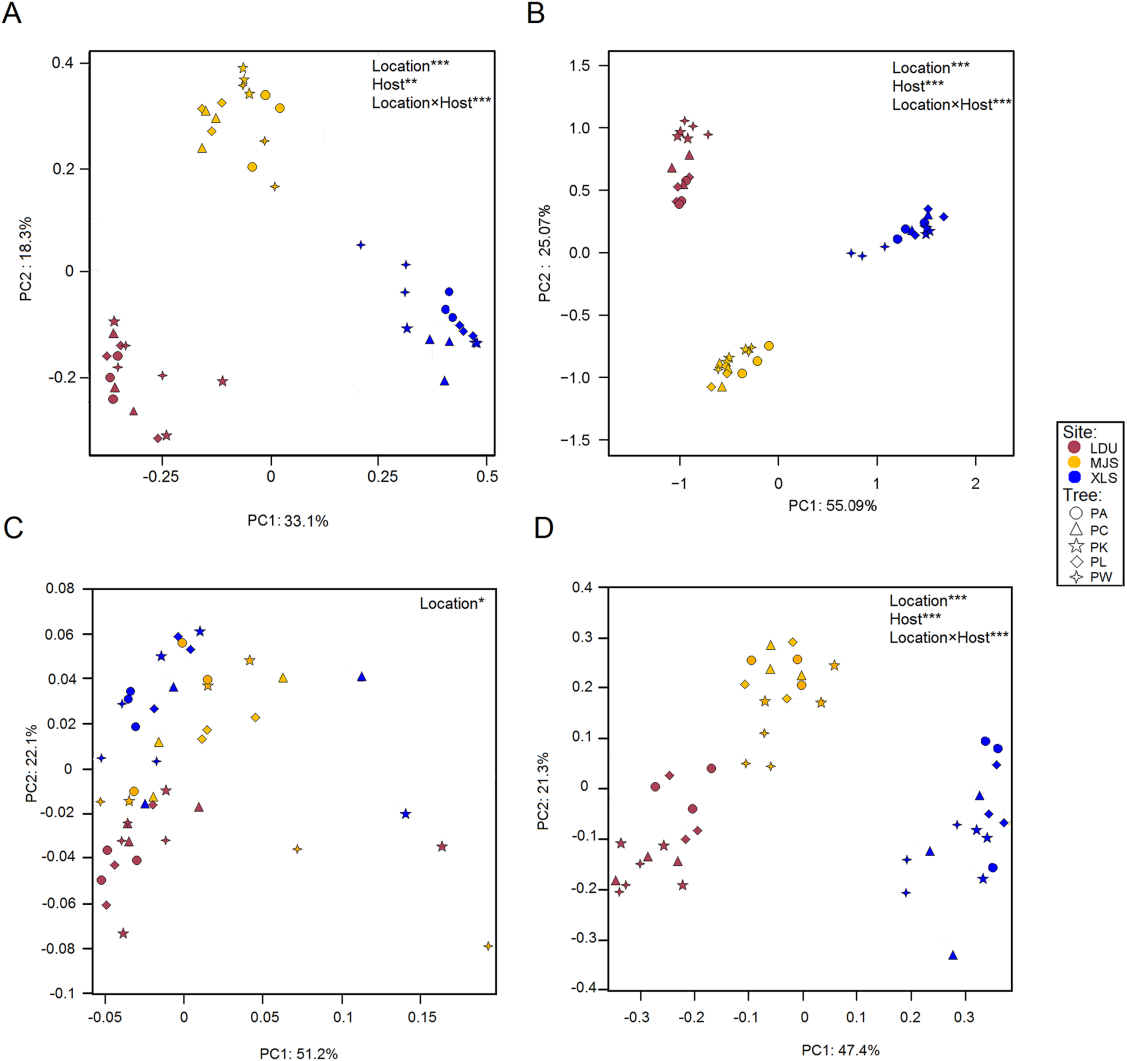
Principal Coordinate Analysis (PCoA) plot of OTUs of bacteria (A) and fungal (B) communities and KEGG orthologs (KOs) (C) and functional guilds (D) based on the Bray–Curtis distance matrix. The symbols are as follows: circles = PA; triangles = PC; pentagram = PK; diamonds = PL; four-angle star = PW. Full name of each plant species is reported into the text. Symbol colors correspond to the different study sites as identified in Fig. 1 (wine red = LDU: Yuzhong campus of Lanzhou University in Lanzhou city; yellow = MJS: a site in the Xinglongshan forest in Majiasi; blue = XLS: Xiaolongshan forest farm in Tianshui City). The values are means of three replicate samples for each host type. Results of the PERMANOVA are given in the higher right of each panel: *p < 0.05; **p < 0.01; ***p < 0.001.

Differences in the microbial OTUs datasets both for the bacterial (Fig. 3A) and fungal (Fig. 3B) communities between all samples were visualized in a PCoA plot, where a clear separation of samples attributed to their geographic locations was evidenced. The significance of these differences was proven by PERMANOVA (Table S4). PERMDISP test demonstrated that variance is homogeneous in different sample groups for bacterial community, bacterial KO, fungal community, fungal guilds (*p* > 0.05) similar in all groups (Table S5). Leaves sampled as little as 14 km apart were found to harbor significantly different taxonomical communities (between LDU and MJS), and the geographic variability on same tree species exceeded the variability in community composition between different trees sampled at one site. Regarding the functional datasets of 16S (Fig. 3C), there is no clear cluster by the geographic location than there was by the taxonomic composition. But the functional composition of the fungal assemblage via FUNGuild designations appears to be more closely tied to the geographical location (Fig. 3D). It could be that this clustering phenomenon is caused by the shortage of the software itself. Because the FUNGuild is a tool of functional prediction which is a simple way to sort large sequence pools into ecologically meaningful categories, in an organism trophic mode not functional gene. Unlike Tax4Fun, the FUNGuild-based methods are inefficient to predicate various functional genes of microorganisms due to the technical shortage. The shortage of FUNGuild can be improved by metagenomic analysis, which is the key to know more about the energy and material metabolism of microbes and communities. But it is less cost-ineffective than FUNGuild, a tool that can be used to taxonomically parse fungal OTUs by the ecological guild independent of the sequencing platform or the analysis pipeline.

### Venn diagrams of taxonomical OTUs and functional groups

**Figure 4:**
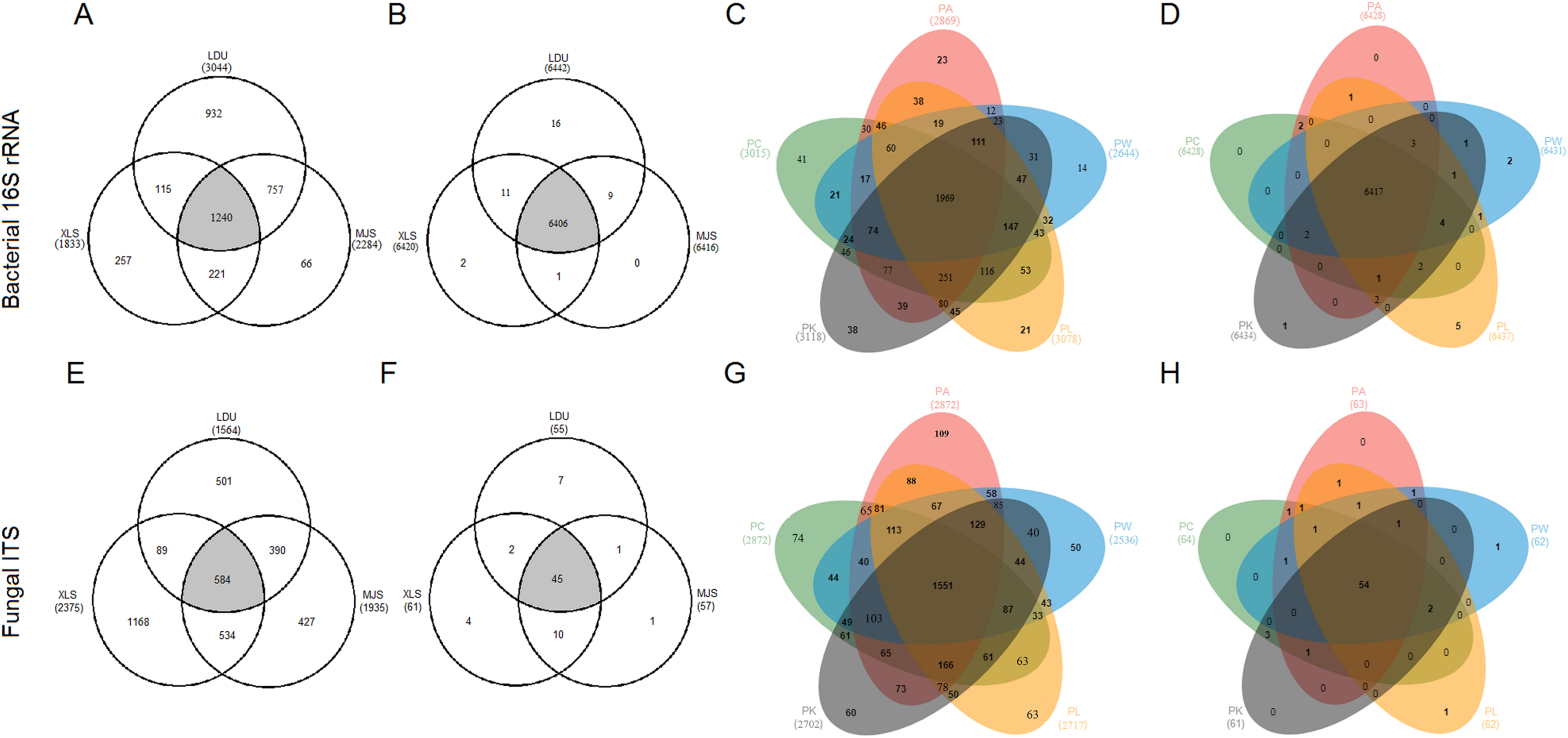
The extent of overlap of phyllosphere microbiome among three locations (A), (B), (E), (F) and among five tree species (C), (D), (G), (H), respectively. At the level of (A), (C), (E), and (G) OTUs clustered at 3% difference; (B), (D), (F), and (H) functional KOs by Tax4Fun and guilds by FUNGuild. The abbreviations of study sites and plant species are as follows: LDU, Yuzhong campus of Lanzhou University in Lanzhou city; MJS, a site in the Xinglongshan forest in Majiasi; XLS, Xiaolongshan forest farm in Tianshui City. PA, *Picea abies*; PC, *Picea crassifolia*; PK, *Picea koraiensis*; PL, *Picea likiangensis var. rubescens*; PW, *Picea wilsonii*.

To evaluate the distribution of OTUs and functional groups among the different sampling locations, Venn diagrams were constructed (Figs. 4A, 4B, 4E and 4F). This showed that 40.74%, 67.65%, and 54.29% of the bacterial OTUs (Fig. 4A) and 37.3%, 24.6%, and 30.2% of the fungal OTUs (Fig. 4E) were common (area in gray) to the three locations (LDU, XLS, and MJS, respectively). However, the proportion of the overlapped functional groups was much higher than those shared by OTUs. In 16S datasets, the overlapped KOs were 99.44% (LDU), 99.78% (XLS), and 99.84% (MJS), respectively (Fig. 4B). Similarly, the overlapped functional guilds were 81.8% (LDU), 73.8% (XLS), and 78.9% (MJS), respectively (Fig. 4F). Among the five-tree species, the Venn Diagrams showed that 67.11% ± 4.62% of the bacterial OTUs (Fig. 4C) and 56.73% ± 2.96% fungal OTUs (Fig. 4G) were shared, respectively. While the overlapped bacterial KOs was up to 99.77% ± 0.06% (Fig. 4D), and the overlapped functional guilds was up to 86.56% ± 1.57% (Fig. 4H).

### Indicator species analysis

Indicator species analysis identifies the bacterial and fungal taxa that are significantly unique to each location. A total of 54 bacterial classes and 58 fungal orders were found with a significant preference (*q* < 0.05) for the three sampling sites, respectively (Tables S6 and S7). In 16S datasets, the 54 classes represented 2.36% of the total bacterial sequences. In ITS datasets, the 58 orders represented 4.09% of the total fungal sequences. There were 20, 12, and 40 indicator fungal taxa in LDU, MJS, and XLS, respectively (Fig. S2B). And there were 53, eight, and five indicator bacterial taxa in LDU, MJS, and XLS, respectively (Fig. S2A). Nearly all the relative abundance of the indicator taxa was low; except two taxa (the classes of Acidobacteriia and Gemmatimonadetes) were more than 1% in bacterial community (Fig. S2A) and two taxa (the orders of Sebacinales and Pezizales) were more than 2% in fungal community (Fig. S2B). Taxonomic names such as “other” and “unidentified” (has no assignment of affiliation at the relevant taxonomic level) were not shown in the bubble plot (Fig. S2).

## Discussion

Our results demonstrate that both geographic locations and host tree species were determinants of the microbial community structure, with the former being significantly more dominant. That is, the effect of the geographic location was probably large enough to cover the influence on the phyllosphere microbiota by the host tree species. The communities on *Arabidopsis thaliana* from a certain site were more similar to those of other plant species from the same site than to *A. thaliana* collected from a different site (Knief et al., 2010). Geographic locations will cause a variation of diverse biotic and abiotic factors between and within the different sampling sites. On the one hand, the geographical location is always accompanied by the site-dependent properties and/or seasonal differences, which can cause changes in the regional species pool. The composition of the pool of colonizing microbiota can be an important structuring factor of the microbiota assembly on the host plant (Callens et al., 2018). And the pool continues to contribute on the microbial community composition. Regarding the *Methylobacterium* community from *A. thaliana* leaves, there is a lower effect of the pure factor plant species contribution compared with the site factor (Knief et al., 2010). On the other hand, the host tree per se is also affected by the contemporary environmental (biotic) factors. Individual trees can alter their development and physiology depending on different environmental conditions (Miner et al., 2005; Nicotra et al., 2010).

The variations in microbial communities stated above refer to microbial taxonomical compositions. Our findings suggest that phyllosphere microbial communities of the differing taxonomical composition are functionally similar. The increase or decrease of individual species will not affect the entire function of the microbial community. The species that are similar taxonomically and morphologically can accomplish many of the equal ecological and physiological functions through their own genetic adaptabilities (Fonseca & Ganade, 2001). The redundant species hypothesis suggests that most species are redundant in their roles, which is known as species “functional redundancy” (Walker, 1992; Lawton & Brown, 1993; Allison & Martiny, 2008). As a result, the species of the same functions will lead to spurious taxonomically differences within a microbial community. The phyllosphere ecosystem function is conserved while species richness varies. This can partly be explained as a result of the initial microbial seed pool plus historical assembly contingencies, for which geographic distance is often used as a measure. We propose future work to explore the assumption of “functional redundancy” for microbial communities in different ecosystems, such as in complex system like the soil-plant-atmosphere continua or aquatic ecosystems.

As indicator species with different indicator values among the various sampling sites, some of them have the same ecosystem functions. To this date, our knowledge about the metabolic properties of members of class-level clades is derived from several closely related cultivated strains. The type strains of the class Holophagae were isolated from anoxic environments (Fukunaga & Ichikawa, 2014). The members of the class Chthonomonadetes, such as *Chthonomonas calidirosea*, is able to metabolize a wide range of mono- and disaccharides as well as branched polysaccharides (Vyssotski et al., 2011). Anaerolineae-type clones have been found frequently within various ecosystems, the organisms within the group have been thought to be ubiquitous and to play important roles in these ecosystems (Yamada et al., 2006). All cultured members of the class are non-motile and can utilize sugars and polysaccharides such as starch, pectin, and xylan (Podosokorskaya et al., 2013). The members from the class Coriobacteriia are also non-motile bacteria (Göker et al., 2010; Gupta et al., 2013). The non-motile property seems to be adverse for the microbes to survive in this habitat. Motility is well established as an important epiphytic fitness factor of plant colonizing microbes, such as *Pseudomonas* (Haefele & Lindow, 1987), and the phylum Bacteroidetes, which is large and diverse, with rapid gliding motility associated with many genera and species (McBride & Zhu, 2013). Characterization of uncultivated groups within Anaerolineae is essential to better understand the metabolic and lifestyle complexity, and epiphytic fitness of this class. We can see that most of the phyllosphere microbes mentioned above based upon their ability to grow via a wide range of mono- and disaccharides as well as branched polysaccharides. Phyllosphere is an oligotrophic environment, in which saccharides are the main source of carbon and energy for heterotrophic microorganisms (Vorholt, 2012). In addition to the saccharides, carbon sources in the leaf surfaces such as one-carbon (C_1_) compounds are also prominent for the phyllosphere microbes. For example, the phylum Planctomycetes present on the leaf surface, harbor a plethora of genes encoding C_1_ compounds metabolism (Youssef & Elshahed, 2014). Most of the C_1_ compounds in the plants are plant-derived metabolic substrates. For instance, methanol is a by-product of cell wall metabolism by pectin methyl esterases (Nemecek-Marshall et al., 1995). Another C_1_ compound that is postulated to be produced in trace amounts by plants is photochemically formed methane (Keppler et al., 2006). Phyllosphere microbes use these as carbon sources for survival (Vorholt, 2012). Some phyllosphere epiphytic microbes are methanogens (Poulsen et al., 2013). For example, some isolates affiliated with the class Thermoplasmata are methanogens, such as archaeon Kjm51a. It is likely to produce methane by the hydrogen-dependent reduction of methanol, which suggested that archaeon Kjm51a is a methanol-reducing hydrogenotrophic methanogen (Iino et al., 2013). The methane that produced by methanogens may also be taken up by other coexisting microorganisms in this niche. There is evidence that carbon source phenotyping of bacteria is frequently used to determine the overlap in substrate utilization among strains as a measure of their potential to coexist (Wilson & Lindow, 1994). The methylotrophic methanogenic lifestyle seems to be very suitable for survival on leaves in which nutrients are scarce and C_1_ compounds are valuable. Thus far, there is no isolation of Parcubacteria. The genomes of all previously studied *Parcubacteria* bacteria have respiratory and fermentative capacities as well as nitrogen and fatty acid metabolisms. Nonetheless, Parcunitrobacteria appears to be unable to synthesize some essential metabolites. Parcunitrobacteria is still known to depend on other organisms (or the community) for many basic building blocks (Castelle et al., 2017). This suggested a coexisting lifestyle for the microbiota establishment of phyllosphere microbial communities. Nitrogen fixation by phyllosphere bacteria has been reported. The globally distributed genus *Nitrospira* (belong to the class Nitrospira) represents the most diverse known group of nitrite-oxidizing bacteria (Daims et al., 2015). Nitrospira members are key components of nitrogen-cycling microbial communities (Lücker et al., 2010). In Thaumarchaeota, genes encoding a homologue of the ammonium-monooxygenase enzyme have been discovered, which is the key enzyme involved in the oxidation of ammonia. Their ability to oxidize ammonia has radically changed the perception of nitrification and global nitrogen cycling in general (Stieglmeier, Alves & Schleper, 2014). All of the taxa discussed above seem to have a propensity for phyllosphere habitat.

Aside from requiring carbon and nitrogen, phyllosphere bacteria need to face the extreme conditions. Microorganisms living on this surface are exposed to a stress consisting of periodic desiccation, moderately high temperatures, high levels of UV radiation, and in some cases high alkalinity (Finkel et al., 2011). Species of Gemmatimonadetes are well adapted to not only arid but also oligotrophic conditions (DeBruyn et al., 2011; Pašić et al., 2010). Some indicator species are associated with stochastic events. For example, the phylum Fibrobacteres, were proven to contain a large amount rumen bacteria. These taxa occur in the intestines of ruminants and nonruminant herbivores and in omnivorous animals (Rosenberg, 2014).

Most fungi within the fungal orders, Ustilaginales (Barr, 1980), Platygloeales (Moore, 1990), Exobasidiales (Begerow, Bauer & Oberwinkler, 2002), Spizellomycetales (Martínez-Espinoza, García-Pedrajas & Gold, 2002), Diaporthales (Rossman, Farr & Castlebury, 2007), and Phaeomoniellales (Chen et al., 2015), are plant pathogens. Caliciales (Holien, 1996), Lichinales (Schultz, Arendholz & Büdel, 2001), and Verrucariales (Gueidan, Roux & Lutzoni, 2007) are lichenized fungi. Most members of Lulworthiales (Kohlmeyer, Spatafora & Volkmann-Kohlmeyer, 2000), Melanosporales (Marin-Felix et al., 2018), and Amylocorticiales (Binder et al., 2010) are undefined saprotrophic fungi. Further, some different indicator taxa in one location always have the same functions. Entylomatales (Begerow, Stoll & Bauer, 2006) and Venturiales (Zhang et al., 2011), are indicators strongly associated with XLS, of which the best-known members are identified as plant pathogens. Mycorrhizal associations are established by the majority of Boletales (Binder & Hibbett, 2006), Pezizales (Danielson, 1984), and Thelephorales (Hibbett, 2006) and they are identified as indicators for LDU.

In conclusion, we observed that the geographical location varies microbial taxonomical compositions in spruce phyllosphere, rather than does the community functional structure. The variation of microbial taxonomy in phyllosphere resulted from the regional seed pool in different geographical locations. Host plant species had both genotypic and phenotypic effects on the microbial communities, in which plant phenotypic traits eventually shape its microbiota due to the community assembly based on the functional types. If the geographic location-governed factors do not cause variations in plant phenotypic traits, the microbial community functional structure will not vary.

## Supplemental Information

10.7717/peerj.7376/supp-1 Supplemental Information 1 OTU tables for both bacterial and fungal reads.

10.7717/peerj.7376/supp-2 Supplemental Information 2 Diversity metrics across various samples.

10.7717/peerj.7376/supp-3 Supplemental Information 3 Results of the two-way ANOVA on the alpha diversity measures. Signif. codes: 0 ‘***’ 0.001 ‘**’ 0.01 ‘*’ 0.05.

10.7717/peerj.7376/supp-4 Supplemental Information 4 Results of the PERMANOVA on the Microbial Taxonomical Composition and Community Functional Structure. Signif. codes: 0 ‘***’ 0.001 ‘**’ 0.01 ‘*’ 0.05.

10.7717/peerj.7376/supp-5 Supplemental Information 5 Results of the PERMDISP on the Microbial Taxonomical Composition and Community Functional Structure.

10.7717/peerj.7376/supp-6 Supplemental Information 6 Result of the indicator species analysis in 16S datasets.

10.7717/peerj.7376/supp-7 Supplemental Information 7 Result of the indicator species analysis in ITS datasets.

10.7717/peerj.7376/supp-8 Supplemental Information 8 Heatmap of abundant OTUs abundance. Replicates and OTUs were clustered according to the Bray-Curtis distances. Sample names are coded as the abbreviations of study sites plus the abbreviations of plant species. A total of 19 and 24 abundant OTUs were identified in 16S and ITS datasets, respectively. There are one and four unclassified OTUs at phylum (in 16S datasets) and order (in ITS datasets) level. The abbreviations of study sites and plant species are as follows: LDU, Yuzhong campus of Lanzhou University in Lanzhou city; MJS, a site in the Xinglongshan forest in Majiasi; XLS, Xiaolongshan forest farm in Tianshui City; PA, *Picea abies*; PC, *Picea crassifolia*; PK, *Picea koraiensis*; PL, *Picea likiangensis var. rubescens*; PW, *Picea wilsonii*.

10.7717/peerj.7376/supp-9 Supplemental Information 9 Bacterial classes (A) and fungal orders (B) identified as indicator taxa significantly (*q* < 0.05) associated with different locations. The size of each circle defines the association strength (indicator value) of a particular taxa with the different location, such as 0–0.25: not characteristic; 0.25–0.5: weakly characteristic; 0.5–0.75: characteristic; 0.75–1.0: strongly characteristic. The bars represent the cumulative relative abundance of each indicator taxa in all the samples.

## Additional Information and Declarations

### Data Availability

The following information was supplied regarding data availability: The phyllosphere and metagenome raw sequence reads are available in the NCBI Trace Archive and the NCBI Sequence Read Archive repositories (BioProject PRJNA506625).
