# Supplementary material for: Microbial taxonomical composition in spruce phyllosphere, but not community functional structure, varies by geographical location
Source: PeerJ. 2019 Jul 19;7:e7376. doi: 10.7717/peerj.7376 (PMC6644631; doi:10.7717/peerj.7376)

Supplemental Figure 1. Heatmap of abundant OTUs abundance. Replicates and OTUs were clustered according to the Bray-Curtis distances. A total of 20 and 24 abundant OTUs were identified in 16S (A) and ITS (B) datasets, respectively. There are one and four unclassified OTUs at phylum (in 16S datasets) and order (in ITS datasets) level

A

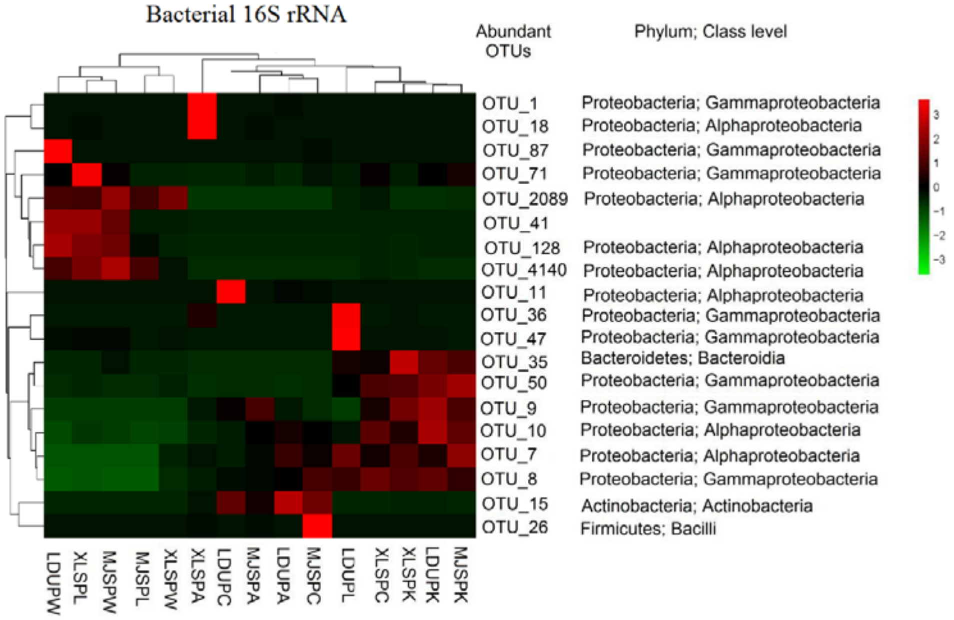

B

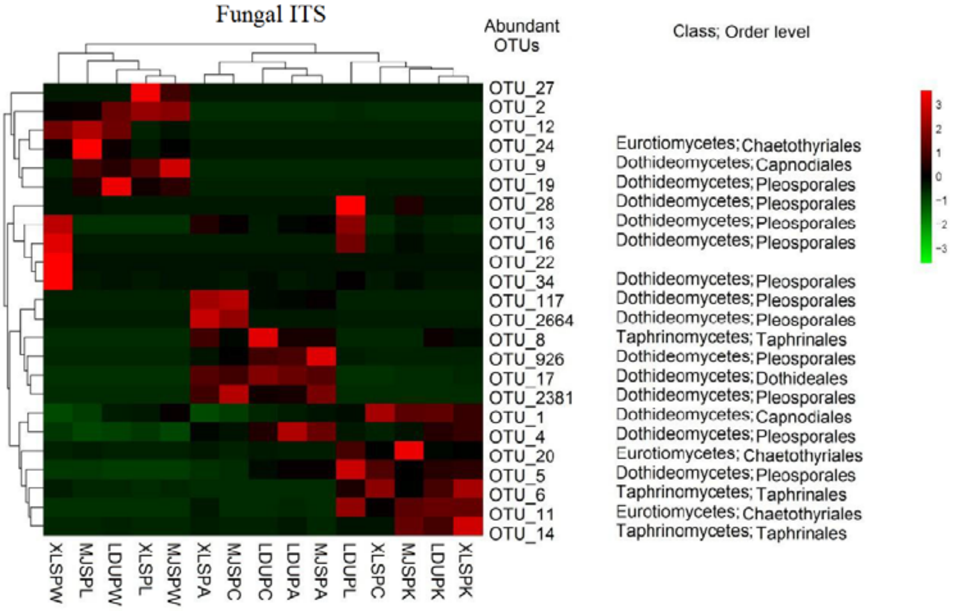

Supplement: Supplemental Information 8 — Sample names are coded as the abbreviations of study sites plus the abbreviations of plant species. A total of 19 and 24 abundant OTUs were identified in 16S and ITS datasets, respectively. There are one and four unclassified OTUs at phylum (in 16S datasets) and order (in ITS datasets) level. The abbreviations of study sites and plant species are as follows: LDU, Yuzhong campus of Lanzhou University in Lanzhou city; MJS, a site in the Xinglongshan forest in Majiasi; XLS, Xiaolongshan forest farm in Tianshui City; PA, Picea abies; PC, Picea crassifolia; PK, Picea koraiensis; PL, Picea likiangensis var. rubescens; PW, Picea wilsonii. [file peerj-07-7376-s008.pdf]
