# Supplementary material for: Microbial taxonomical composition in spruce phyllosphere, but not community functional structure, varies by geographical location
Source: PeerJ. 2019 Jul 19;7:e7376. doi: 10.7717/peerj.7376 (PMC6644631; doi:10.7717/peerj.7376)
Supplement: Supplemental Information 9 — The size of each circle defines the association strength (indicator value) of a particular taxa with the different location, such as 0–0.25: not characteristic; 0.25–0.5: weakly characteristic; 0.5–0.75: characteristic; 0.75–1.0: strongly characteristic. The bars represent the cumulative relative abundance of each indicator taxa in all the samples. [file peerj-07-7376-s009.pdf]

Supplemental Figure 2. Bacterial classes (A) and fungal orders (B) identified as indicator taxa significantly (q<0.05) associated with different locations. The size of each circle defines the association strength (indicator value) of a particular taxa with the different location, such as 0-0.25: not characteristic; 0.25-0.5: weakly characteristic; 0.5-0.75: characteristic; 0.75-1.0: strongly characteristic. The bars represent the cumulative relative abundance of each indicator taxa in all the samples

A

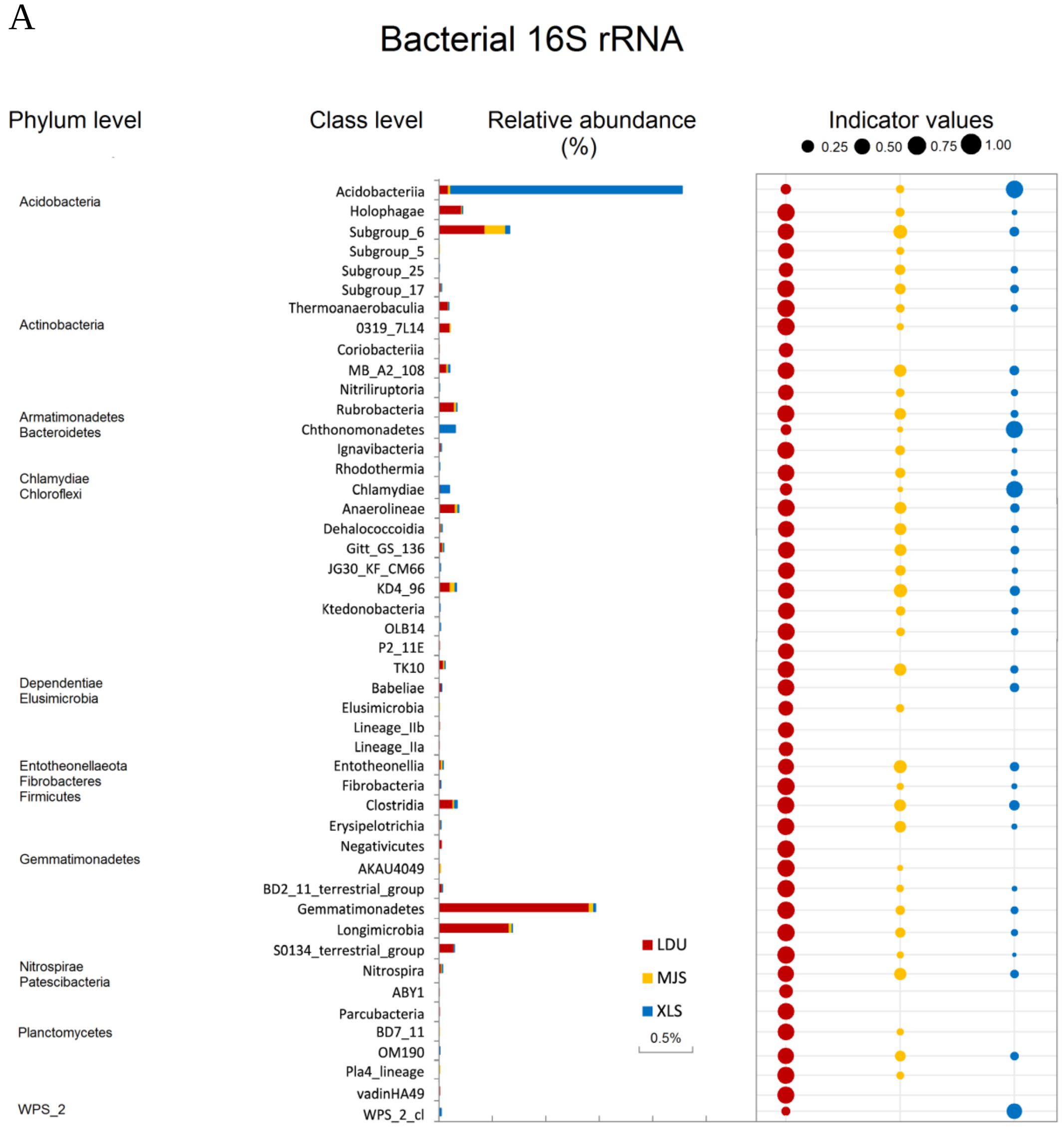

B

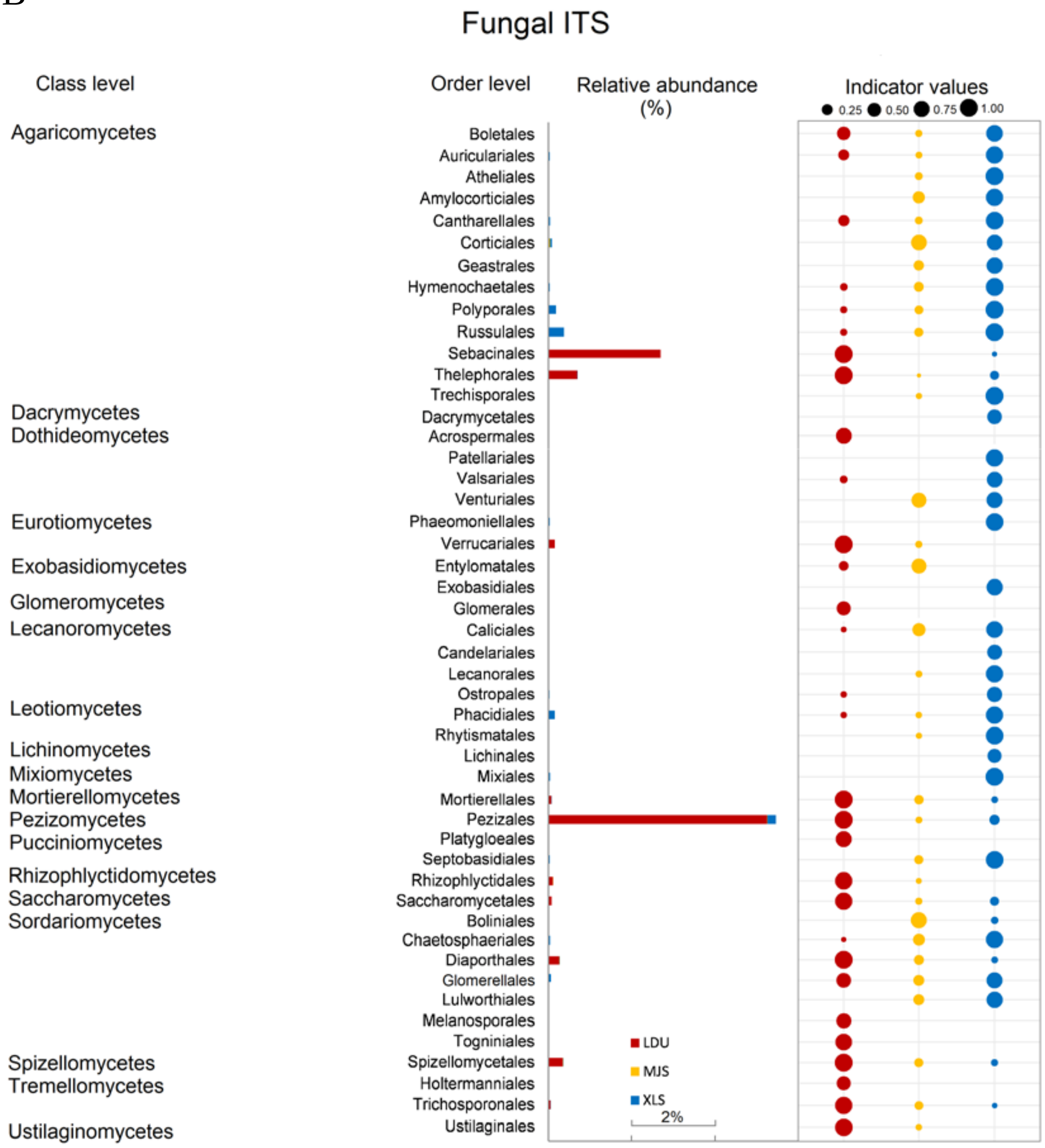

Fungal ITS
Class level
Order level
Relative abundance (%)
Indicator values
0.25
0.50
0.75
1.00
Agaricomycetes
Dacrymycetes
Dothideomycetes
Eurotiomycetes
Exobasidiomycetes
Glomeromycetes
Lecanoromycetes
Leotiomycetes
Lichinomycetes
Mixiomycetes
Mortierellomycetes
Pezizomycetes
Pucciniomycetes
Rhizophlyctidomycetes
Saccharomycetes
Sordariomycetes
Spizellomycetes
Tremellomycetes
Ustilaginomycetes
Boletales
Auriculariales
Atheliales
Amylocorticiales
Cantharellales
Corticiales
Geastrales
Hymenochaetales
Polyporales
Russulales
Sebacinales
Thelephorales
Trechisporales
Dacrymycetales
Acrospermales
Patellariales
Valsariales
Venturiales
Phaeomoniellales
Verrucariales
Entylomatales
Exobasidiales
Glomerales
Caliciales
Candelariales
Lecanorales
Ostropales
Phacidiales
Rhytismatales
Lichinales
Mixiales
Mortierellales
Pezizales
Platygloeales
Septobasidiales
Rhizophlyctidales
Saccharomycetales
Boliniales
Chaetosphaeriales
Diaporthales
Glomerellales
Lulworthiales
Melanosporales
Togniniales
Spizellomycetales
Holtermanniales
Trichosporonales
Ustilaginales
LDU
MJS
XLS
2%
